# Supplementary material for: The E2F Pathway Score as a Predictive Biomarker of Response to Neoadjuvant Therapy in ER+/HER2− Breast Cancer
Source: Cells. 2020 Jul 8;9(7):1643. doi: 10.3390/cells9071643 (PMC7407968; doi:10.3390/cells9071643)
Supplement: Supplementary file 1 [file cells-09-01643-s001.pdf]

Table S1. Overlaps of member genes of the E2F and other pathway gene sets.

Gene Set Name	Total Genes	Genes in Overlap
E2F_TARGETS	200	200
G2M_CHECKPOINT	200	73 (36.5%)
MYC_TARGETS_V2	58	12 (20.7%)
MYC_TARGETS_V1	200	35 (17.5%)
DNA_REPAIR	150	15 (10%)
MITOTIC_SPINDLE	199	19 (9.5%)
MTORC1_SIGNALING	200	14 (7.0%)
UNFOLDED_PROTEIN_RESPONSE	200	6 (3.0%)

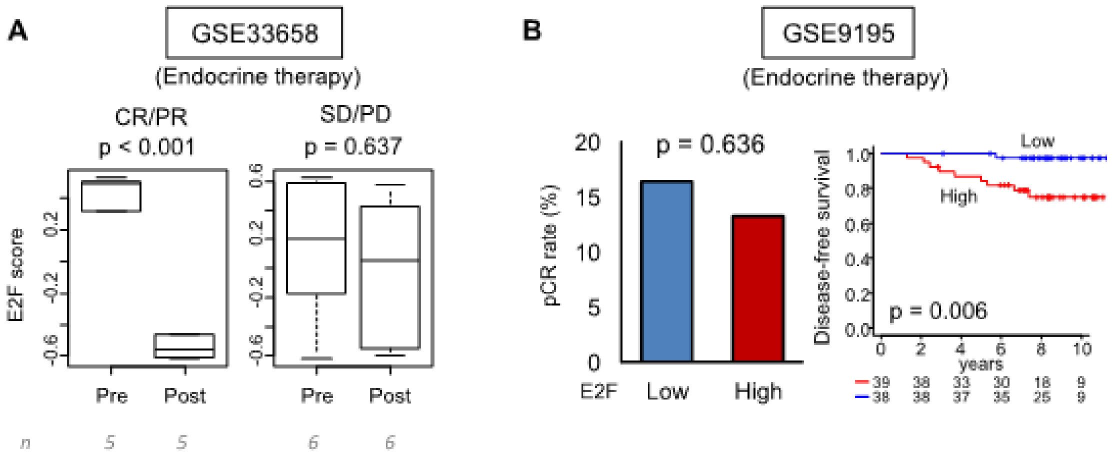

Figure S1. Association between the E2F pathway score and response to endocrine therapy. **(A)** Boxplots depict tumor E2F scores before (*pre*) and after (*post*) treatment for patients of the GSE33658 cohort who had complete or partial response (*CR/PR: n* = 10), or steady or progressive disease (*SD/PD; n* = 12) following treatment as per RECIST criteria. Tukey type boxplots demonstrate median and inter-quartile level values, and p values are calculated by ANOVA test. Group sizes (*n*) are noted underneath. **(B)** Patients of the GSE9195 cohort (*n* = 77) with high or low pre-treatment tumor E2F scores are com-pared for achievement of pathological complete response (*pCR*) to treatment and dis-ease-free survival. The median value of the E2F core is used to classify patients into high and low groups. Rate of *pCR* and Kaplan-Meier survival curves are respectively compared with Fisher exact and logrank tests.

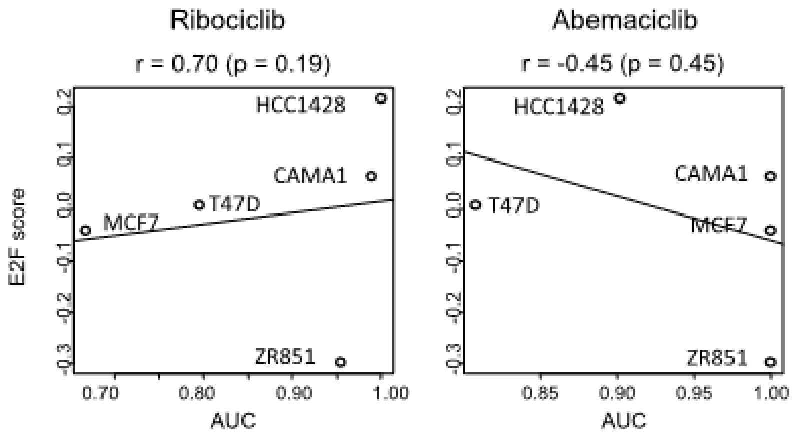

Figure S2. Association in human breast cancer cell-lines between E2F pathway score and sensi-tivity to cyclin-dependent kinase inhibitors. Sensitivity to inhibitors ribociclib or ber-naciclib, measured in area under curve (*AUC*) units in drug sensitivity assays, is plot-ted against E2F score for five cell-lines. Spearman correlation statistics are depicted.
